# Environmental chemical exposures and mental health outcomes in children: a narrative review of recent literature

**DOI:** 10.3389/ftox.2023.1290119

**Published:** 2023-11-30

**Authors:** Ashley A. James, Katherine L. OShaughnessy

**Affiliations:** ^1^ United States Environmental Protection Agency, Office of Children’s Health Protection, Regulatory Support and Science Policy Division, Washington, DC, United States; ^2^ Oak Ridge Institute for Science Education, Oak Ridge, TN, United States; ^3^ United States Environmental Protection Agency, Public Health Integrated Toxicology Division, Center for Public Health and Environmental Assessment, Research Triangle Park, NC, United States

**Keywords:** children’s mental health, behavioral health, environmental justice, environmental exposure, toxicity, heavy metals, endocrine disrupting chemicals, pesticides

## Abstract

**Background:** Mental health is an important factor for children’s overall wellbeing. National health statistics show that millions of children are diagnosed with mental health disorders every year, and evidence from studies on chemical pollutants like lead and bisphenols indicate that environmental exposures are linked to mental health illnesses in youth. However, the relationship between children’s mental health and the environment is not well understood. This paper aims to review recent literature on prenatal and/or childhood environmental chemical exposures and mental health problems related to mood, anxiety, and behavior. This work also identifies areas of insufficient data and proposes suggestions to fill the data gaps.

**Methods:** A narrative review was performed by searching Google Scholar and PubMed for literature published in the last 6 years (2017–2022), using search terms related to children, mental health, and environmental chemical exposure. Additional relevant studies were identified by screening the references in these papers.

**Results:** A total of 29 studies are included in this review and results are summarized by chemical category: heavy metals, endocrine-disrupting chemicals, and pesticides. The majority of studies reported positive and significant associations between chemical exposures and child mental health outcomes including internalizing and externalizing behaviors.

**Conclusion:** This review demonstrates that there is a growing body of literature that suggests developmental exposure to some environmental chemicals increases a child’s risk of mood, anxiety, and behavior problems. Future research should expand on these findings to understand cumulative impacts, chemical mixtures, neurotoxic mechanisms, sex differences, and windows of vulnerability.

## 1 Introduction

Children’s mental health is a critical factor of their overall health and wellbeing. Mental health disorders, including mood, anxiety, and behavioral disorders, impact multiple aspects of children’s lives including the development of social skills, academic performance, and physical health. The diagnosis of childhood mental health disorders is also one of the strongest predictors of adult mental health disorders (Fryers and Brugha, 2013). Over the past 2 decades, the prevalence of adverse mental health outcomes among U.S. children has increased (Park-Lee, 2019; Tkacz and Brady, 2021). Based on 2016–2019 surveillance data for children ages 3–17 years living in the United States, 8.9% of children had received a behavior problems diagnosis, 4.4% of children had received a depression diagnosis, and 9.4% of children had received an anxiety diagnosis (Bitsko et al., 2022). Given the inherent gaps in surveillance data, including that many children mental health disorders are undiagnosed or misdiagnosed (Bitsko et al., 2022), these numbers may be underestimates.

The etiology of psychopathology is complex and includes genetic, social, and environmental factors, including exposures to chemicals in the environment. In recent years, the environmental determinants of mental health outcomes have gained increasing attention in multiple scientific disciplines including toxicology, epidemiology, psychology, and psychiatry. For example, there is a growing body of research that indicates air pollution may negatively impact children’s mental health (Brokamp et al., 2019; Roberts et al., 2019; Szyszkowicz et al., 2020; Reuben et al., 2021). Data on anthropogenic climate change and its negative impacts on youth mental health outcomes is also growing (Burke et al., 2018; Van Nieuwenhuizen et al., 2021; Clemens et al., 2022), including the consideration of “climate anxiety,” or anxiety related to the climate crisis and environmental disasters (Wu et al., 2020; Hickman et al., 2021). Further, the emerging discipline of clinical ecopsychology, which seeks to “systematically examine the direct and indirect mental health impacts of climate change, pollution, environmental degradation, and/or destruction of the air, water, and ecosystems,” has shown that environmental crisis and pollution act as stressors and disrupt multiple pathways that can increase a person’s vulnerability to adverse mental health (Thoma et al., 2021). Despite this growing interest, several data gaps related to environmental exposures and child mental health outcomes remain.

Scientists, physicians, and decision-makers need to better understand how environmental pollutants may impact children’s mental health. There are several plausible mechanisms to link environmental chemical exposures to the induction and/or exacerbation of mental health disorders. Chemical pollutants, such as heavy metals and endocrine disruptors, may alter neurotransmitter systems, damage the blood-brain barrier, modify brain gene expression leading to increased vulnerability to mental health disorders (i.e., depression), dysregulate the hypothalamus-pituitary-adrenal axis, reduce neuronal plasticity, and induce oxidative stress and neuroinflammation (Van Den Bosch and Meyer-Lindenberg, 2019; Thoma et al., 2021). The latter three are also consequences of chronic stress, which can have a synergistic effect on mental health outcomes (Van Den Bosch and Meyer-Lindenberg, 2019; Thoma et al., 2021).

Neurodevelopment is a protracted process that occurs from early embryogenesis and continues until at least 21 years of age (Stiles and Jernigan, 2010). Therefore, children are vulnerable to environmental exposures for years, and any perturbation of normal neurodevelopment could hold lifelong consequences (Stiles and Jernigan, 2010). Furthermore, children have limited ability to handle stress due to immature coping mechanisms, and thus have increased vulnerability to life stressors that can impact brain structure and function (Thoma et al., 2021). For example, racial and economic disparities in child mental health outcomes exist. A recent study found that non-Hispanic Black children had higher rates of mental-health related emergency visits than white children (Abrams et al., 2022). Several studies have also demonstrated that children from low-income households have a higher prevalence of mental health disorders than children from middle- and high-income households (Roberts et al., 2007; Melchior et al., 2010; Najman et al., 2010; Acri et al., 2017; Cree et al., 2018). These studies suggest that children living in environmental justice communities may be more at-risk to mental health conditions, given their burden of both chemical and non-chemical stressors. This review aims to assess the landscape of recent literature on prenatal and/or childhood environmental xenobiotic exposures and consequent symptoms related to mood, anxiety, and behavior disorders.

## 2 Materials and methods

This review focuses on symptoms and behaviors related to mood, anxiety, and behavior disorders such as depression, generalized anxiety disorder, and conduct disorder, respectively. Studies that exclusively focused on neurodevelopmental disorders (i.e., attention deficit hyperactivity disorder and autism spectrum disorder), cognition, intelligence quotient, and/or learning disorders were excluded. Experimental animal and adult population studies were also excluded, unless related to a childhood exposure. This review considered exposures from conception until age 21 years, in accordance with the U.S. Environmental Protection Agency’s Policy on Children’s Health (U.S. EPA, 2021).

To include the most recent research, only original research studies from the last 6 years were included, from January 2017 to December 2022. Google Scholar and PubMed were searched using search terms related to children (i.e., “child” or “infant” or “prenatal” or “adolescent”), mental health (i.e., “mental health” or “stress” or “anxiety” or “behavior” or “mood”), and environmental chemical exposure (i.e., “pollution” or “chemical” or “toxins”). Reference lists were also screened for additional relevant studies. Papers were selected based on title and then abstracts were screened. Full texts were then checked according to the inclusion/exclusion criteria, resulting in 29 final papers selected for review. Given that there is limited data on this emerging research area, we chose a narrative review, which is useful for exploring under researched topics (Sukhera, 2022). The approach was not intended to follow systematic review procedures.

## 3 Results

### 3.1 Literature overview

A total of 29 studies were included in this review (Table 1). These results are summarized below by chemical category, with some papers addressing the effects of more than one chemical class. The distribution of papers by chemical class are as follows: 11 papers on heavy metals, 20 on endocrine disrupting chemicals, and 4 on pesticides. Most of the studies (*n* = 22) used a prospective cohort design, with 20 studies being longitudinal birth cohorts. The majority of studies (*n* = 20) assessed prenatal exposure and measured outcomes during early childhood, from 0 to 5 years (*n* = 13), and mid childhood, from 6 to 10 years (*n* = 14). Each study utilized various covariates like maternal age and education, and this information can be found in the original publications. Eleven of the studies were conducted in North America, 7 in Asia, 5 in Europe, 5 in Latin America, and 1 in Oceania (New Zealand). Internalizing behaviors (i.e., anxiety, depression, somatization, withdrawal) and externalizing behaviors (i.e., aggression, impulsivity, conduct disorder) were the most frequently assessed endpoints. The most common measures used to assess these behaviors were the Behavior Assessment System for Children 2nd Edition (BASC-2) (*n* = 9), the Strengths and Difficulties Questionnaire (SDQ) (*n* = 7) and the Child Behavior Checklist (CBCL) (*n* = 7); information regarding these behavioral metrics can be found in Table 2. All findings summarized in this section are statistically significant, unless stated otherwise.

**TABLE 1:** Summary of the literature reviewed.

Citation	Chemical class	Chemical name	Assessment tool	Results
Zeng et al. (2021)	Metals	Lead	The Strengths and Difficulties Questionnaire (SDQ)	Blood Pb level was negatively correlated with NPY, but positively correlated with behavioral symptom scores; while serum NPY levels were negatively associated with behavioral symptom scores
Joo et al. (2018)	Metals	Lead	Child Behavior Checklist (CBCL)	Prenatal Pb associated with increased total behavior scores in preschool males. Early childhood Pb associated with increased total behavior scores in preschool females
Horton et al. (2018)	Metals	Lead, zinc, manganese, mixture	The Behavior Assessment System for Children 2nd Edition (BASC-2)	Infant Pb exposure (measured in deciduous teeth) associated with increased anxiety symptoms in mid childhood
Reuben et al. (2019)	Metals	Lead	Big Five Personality Inventory	Mid childhood blood Pb levels associated with increased internalizing symptoms and general psychopathology in adults
Rokoff et al. (2022)	Metals, Pesticides	Organochlorines, lead, manganese	The Behavior Assessment System for Children 2nd Edition (BASC-2) and Conners’ Rating Scale (CRS)	Prenatal Pb associated with increased 15-year-old anxiety symptoms and psychosomatic symptoms in males. Prenatal Mn associated with increased internalizing symptoms in girls
Rodrigues et al. (2018)	Metals	Manganese	Child Behavior Checklist (CBCL)	Toenail Mn associated with increased total behavioral and externalizing behavior scores in 7–12-year-olds, with a stronger effect for externalizing behavior in boys
Rahman et al. (2017)	Metals	Manganese	The Strengths and Difficulties Questionnaire (SDQ)	Prenatal Mn exposure from drinking water increased the risk of conduct problems in 10-year old children, with a stronger effect in boys
de Water et al. (2018)	Metals	Manganese	functional magnetic resonance imaging (fMRI)	Maternal blood Mn were associated with reduced functional connectivity of brain areas involved in emotional processing and regulation in 6- to 7-year-old children
Kim et al. (2018)	Metals, EDCs	Phthalates, heavy metals, and persistent organic pollutants	Child Behavior Checklist (CBCL)	Prenatal blood Hg associated with and higher internalizing and externalizing scores in Korean toddlers aged 1–2 years. Prenatal MEP associated with increased internalizing scores
Maitre et al. (2021)	Metals	Copper	Child Behavior Checklist (CBCL)	Cu exposure, measured in blood, associated with higher internalizing symptoms in 6- to-11-year-old children
Jedynak et al. (2021)	Metals, EDCs	BPA, phthalates, copper	The Strengths and Difficulties Questionnaire (SDQ)	Prenatal blood Cu associated with decreased externalizing behavior, prenatal urinary MBzP associated with increased externalizing behavior, and prenatal urinary BPA associated with increased externalizing behaviors in children ages 3 to 7
Singer et al. (2017)	EDCs	Phthalates	Infant Behavior Questionnaire (IBQ) Toddler Behavior Assessment Questionnaire (TBAQ)	Weak positive associations with prenatal exposure to multiple phthalates and temperament in infants
England-Mason et al. (2020)	EDCs	Phthalates	Child Behavior Checklist (CBCL)	Prenatal exposure to high molecular weight phthalates indirectly associated with internalizing and externalizing problems through mean diffusivity (MD) of different parts of the brain
Chen et al. (2019)	EDCs	Phthalates	Child Behavior Checklist (CBCL)	Prenatal DEHP exposure associated with increased behavior scores in all categories except somatic complaints in 8-, 10-, and 14-year-olds
Huang et al. (2019)	EDCs	Phthalates	Child Behavior Checklist (CBCL)	Prenatal DEHP exposure associated with CBCL higher delinquent behavior and externalizing problem scores in 8-to 14-year-olds
Colicino et al. (2021)	EDCs	Phthalates	The Behavior Assessment System for Children 2nd Edition (BASC-2)	Prenatal urinary DEHT exposure with increased overall BASC-2 behavioral problems and depression scores in 4- to 6- year-old boys
Philippat et al. (2017)	EDCs	Phthalates, phenols	The Strengths and Difficulties Questionnaire (SDQ)	Prenatal urinary MnBP and MBzP associated with increased internalizing behavior in 3-year-old boys. Prenatal urinary BPA associated with increased internalizing behavior in 3-year-old boys and increased externalizing behavior in 5-year-old boys
Vuong et al. (2021)	EDCs	PFAS	The Behavior Assessment System for Children 2nd Edition (BASC-2)	Prenatal serum PFOS, PFHxS, and PFNA associated with worse externalizing behavior, and PFHxS was associated with worse internalizing behavior at ages 5 and 7 years
Ghassabian et al. (2018)	EDCs	PFAS, phenols	The Strengths and Difficulties Questionnaire (SDQ)	Newborn dried blood spot BPA exposure associated with decreased prosocial behavior difficulties (2nd and 4th quartile only) and newborn PFOS exposure associated with increased conduct and emotional problems at age 7
Luo et al. (2020)	EDCs	PFAS	The Strengths and Difficulties Questionnaire (SDQ)	Prenatal plasma PFNA associated with increased externalizing behaviors at ages 7 and 11 years
Strawn et al. (2022)	EDCs	PBDE	Screen for Child Anxiety Related Emotional Disorders (SCARED)	Prenatal PBDE exposure associated with increased anxiety symptoms in 12-year-olds
Braun et al. (2017)	EDCs	PBDE, BPA	The Behavior Assessment System for Children 2nd Edition (BASC-2)	Prenatal serum BDE-47 exposure associated with increased externalizing behaviors in 2- to 8- year-olds. Prenatal urinary BPA associated with increased externalizing behavior in girls only
Vuong et al. (2017)	EDCs	PBDE	The Behavior Assessment System for Children 2nd Edition (BASC-2)	Concurrent PBDE serum exposure in 8-year-olds associated with increased externalizing symptoms
Shoaff et al. (2019)	EDCs	Phthalates, phenols, parabens	The Behavior Assessment System for Children 2nd Edition (BASC-2)	Sum of 11 antiandrogenic phthalate metabolites collected from adolescent spot urine associated with increased maladaptive behaviors. Null results for exposure to phenols
Stacy et al. (2017)	EDCs	Phenols	The Behavior Assessment System for Children 2nd Edition (BASC-2)	Prenatal urinary BPA increased externalizing behavior in girls (at age 8) and 8-year BPA increased externalizing behavior in boys
Zhang et al. (2022)	EDCs	Phenols	Center for Epidemiologic Studies Depression Scale for Children (CES-DC)	Serum Bisphenol AF exposure associated with increased depressive symptoms in adolescents (7th grade), with males significantly more vulnerable
Rosenquist et al. (2017)	EDCs, pesticides	DDT	The Strengths and Difficulties Questionnaire (SDQ)	Pre- and postnatal serum p,p′-DDE associated with increased conduct problems in 5- and 7-year-olds
Suarez-Lopez et al. (2021)	Pesticides	Organophosphates	Multidimensional Anxiety Scale for Children 2nd Edition (MASC-2) or the Children’s Depression Inventory 2nd Edition (CDI-2)	Decrease in AChE activity, from exposure to organophosphate pesticides, associated with increase depression symptoms of 11–17-year-old adolescents
Furlong et al. (2017)	Pesticides	Pyrethroids	The Behavior Assessment System for Children 2nd Edition (BASC-2)	Prenatal urinary 3-PBA and cis-DCCA associated with increased internalizing and externalizing behaviors respectively

**TABLE 2:** Summary of the behavioral assessments used to quantify mental health in children.

Name	Abbreviation	Scales	Assessor	Count (n)
The Strengths and Difficulties Questionnaire	SDQ	Emotional Symptoms, Conduct Problems, Hyperactivity/Inattention, Peer Relationship Problems, Prosocial Behavior, Total Difficulties Score	Parent, Teacher, or Self-Report for Adolescents	7
The Behavior Assessment System for Children 2nd Edition	BASC-2	Internalizing Problems (Anxiety, Depression, Somatization), Externalizing Problems (Hyperactivity, Aggression, Conduct Disorder), School Problems (Attention Problems, Learning Problems), and Adaptive Skills (Adaptability, Social Skills, Leadership, Study Skills)	Parent, Teacher, or Self-Report for Adolescents	9
Child Behavior Checklist/Korean Child Behavior Checklist	CBCL/KCBCL	Syndrome Scales: Withdrawn/Depressed, Somatic Complaints, Anxious/Depressed, Social Problems, Thought Problems, Attention Problems, Delinquent Behavior, Aggressive Behavior, Internalizing Score, Externalizing Score, Total Problems Score. Diagnostic and Statistical Manual of Mental Disorders Scales: Anxiety, Oppositional Defiant Disorder, Conduct Problems, Somatic Problems, Affective Problems, Attention Deficit Disorder	Parent, Teacher, or Self-Report for Adolescents	7
Big Five Personality Inventory	BFI	Extraversion, Agreeableness, Openness, Conscientiousness, and Neuroticism	Self-Report	1
Conners' Rating Scale	CRS	Oppositional, Cognitive, Problems/Inattention, Hyperactivity, Anxious-Shy, Perfectionism, and Social Problems, ADHD Index Score, a DSM-IV: Inattention Score, and a DSM-IV: Hyperactivity’ Score	Parent, Teacher, or Self-Report for Adolescents	1
Multidimensional Anxiety Scale for Children 2nd Edition	MASC-2	Separation Anxiety/Phobias, Social Anxiety, Obsessions & Compulsions, Physical Symptoms, Harm Avoidance, GAD Index and Inconsistency Index	Parent or Self-Report	1
Children’s Depression Inventory 2nd Edition	CDI-2	Scales: Emotional Problems, Functional Problems. Subscales: Negative Mood/Physical Symptoms, Negative Self-Esteem, Interpersonal Problems, Ineffectiveness	Self-Report	1
The Behavior Rating Inventory of Executive Functioning	BRIEF	Clinical Scales: Inhibit, Shift, Emotional Control, Initiate, Working Memory, Plan/Organize, Organization of Materials, Monitor. Validity scales: Inconsistency and Negativity. Global Executive Composite	Parent or Teacher	1
Screen for Child Anxiety Related Emotional Disorders	SCARED	Generalized Anxiety, Separation Anxiety, Social Anxiety, Panic or Somatic Symptoms, School Avoidance	Parent or Self-Report	1
Infant Behavior Questionnaire	IBQ	Approach, Vocal Reactivity, High Intensity Pleasure, Smiling and Laughter, Activity Level, Perceptual Sensitivity, Sadness, Distress to Limitations, Fear, Falling Reactivity/Rate of Recovery from Distress, Low Intensity Pleasure, Cuddliness, Duration of Orienting, Soothaability	Parent	1
Toddler Behavior Assessment Questionnaire	TBAQ	Activity level, Anger, Social Fear, Interest, Pleasure	Parent	1
Center for Epidemiologic Studies Depression Scale for Children	CES-DC	One scale with 20 items designed to specifically measure for depression	Self-Report	1

### 3.2 Heavy metals

Heavy metals are naturally occurring chemical elements that have a vast range of industrial uses but can be extremely toxic to several organ systems, including the central nervous system. Heavy metal applications include agriculture (e.g., fertilizer), technology (e.g., electronics), home goods (e.g., cookware) and manufacturing (e.g., automobile), which result in multiple possible sources of exposures. Documented exposure routes in humans include inhalation, ingestion, and dermal absorption (Al-Osman et al., 2019).

#### 3.2.1 Lead

Lead (Pb) is one of the most well-established neurodevelopmental toxicants with no known safe level of exposure (U.S. EPA, 2013). Sources of Pb exposure for children include toys, dust, residential lead paint, drinking water, and soil (Njati and Maguta, 2019; Wilson et al., 2022). Pb accumulates in bone, likely due to its similar atomic properties to calcium. During pregnancy, Pb harbored in bone can be released into the bloodstream due to normal maternal bone remodeling, which results in elevated maternal Pb concentrations and consequently increased fetal exposure (reviewed in Goyer, 1996). Hypothesized mechanisms for Pb induced neurotoxicity include oxidative stress and neuronal cell death (Nemsadze et al., 2009; Ramírez Ortega et al., 2021). A study of Chinese pre-school students also found that electronic waste Pb exposure may decrease serum Neuropeptide Y, and that Neuropeptide Y may mediate a positive association between blood Pb level and behavioral deficits. However, this study was cross-sectional and thus could not establish causality (Zeng et al., 2021).

In the U.S., a birth cohort study found a positive association between prenatal Pb exposure (via umbilical cord blood) and BASC-2 anxiety score in 15-year-old adolescents, indicating increased anxiety symptoms (Rokoff et al., 2022). The study found a positive association between prenatal Pb and BASC-2 psychosomatic scores, with a stronger effect in males (Rokoff et al., 2022). A Korean birth cohort study found sex differences as well, with higher prenatal blood Pb levels increasing total CBCL behavior scores more in 5-year-old males, and higher childhood blood Pb levels (measured at ages 2,3, and 5) increasing total CBCL behavior scores more in 5-year-old females (Joo et al., 2018). When analyzing deciduous teeth to estimate developmental Pb exposure, a birth cohort study in Mexico associated increased dentine Pb at 12 months old with increased BASC-2 anxiety symptoms at 8–11 years (Horton et al., 2018).

Only one cohort study, conducted in New Zealand, reported results on early life Pb exposure and adult mental health (up to age 38 years) (Reuben et al., 2019). The study found that higher childhood Pb exposure, as determined by Pb blood concentrations measured at 11 years of age, was associated with increased internalizing symptoms and increased general psychopathology. However, this is a single study conducted in the 1970s, so these results may not be generalizable and should be replicated in other populations. In total, Pb is a recognized neurotoxicant. In addition to established effects like reduced intelligent quotient scores (IQ) (Lanphear et al., 2005; McFarland et al., 2022), these recent epidemiological studies also suggest that increased Pb exposure are correlated to negative mental health consequences in children.

#### 3.2.2 Manganese

Manganese (Mn) is essential for brain function, but neurotoxic in excess. Children can be exposed to Mn prenatally (i.e., maternal blood), and via air pollution, food, and drinking water (Krachler et al., 1999; Rahman et al., 2017; Rodrigues et al., 2018). Inhaled Mn can cross the blood brain barrier, accumulate in the brain, and alter synaptic mechanisms (Davis, 1999; Aschner and Dorman, 2006) Mn has also been shown to impair astrocyte function, disrupt myelination, and disrupt dopamine neurotransmission (Normandin and Hazell, 2002). Using functional magnetic resonance imaging (fMRI), a birth cohort study in Mexico found that higher levels of Mn in maternal blood were associated with reduced functional connectivity of brain areas involved in emotional processing and regulation in 6- to 7-year-old children (de Water et al., 2018).

A cross-sectional study in Brazil assessed behavior in 7- to 12-year-old children residing near a ferro-manganese alloy plant, which resulted in increased exposure to airborne Mn (Rodrigues et al., 2018). The team used toenail clippings to assess long-term (7–12 month) Mn exposure and found significant associations between increased Mn exposure and increased total SDQ behavioral and externalizing behavior scores; this indicates that high Mn correlated to worsening behavioral symptoms. In Bangladesh, Rahman et al. (2017) found increased prenatal Mn exposure from drinking water increased the risk of conduct problems in 10-year-old children, as measure by the SDQ. Both studies saw more pronounced effects for externalizing behavior symptoms in boys (Rahman et al., 2017; Rodrigues et al., 2018). Whereas in the U.S., Rokoff et al. (2022) observed a positive association between prenatal Mn exposure, measured in umbilical cord blood, and internalizing symptoms in girls, at ages 8 years (measured by the Conners’ Rating Scale) and 15 years (measured by BASC-2), but saw null results in boys.

Like Pb, Mn is a well-recognized neurotoxicant. A large portion of the Mn literature focuses on adult neurotoxicity, as Mn excess can cause a Parkinson-like syndrome, including memory and motor deficits (Kwakye et al., 2015). The few studies presented here suggests that Mn excess may also contribute to mental health and behavioral problems in children. Further studies examining these neurological effects during development are warranted.

#### 3.2.3 Copper

Copper (Cu) is an essential mineral for brain development but neurotoxic at high concentrations. Children can be exposed to Cu during gestation, and from sources such as drinking water (e.g., Cu pipes), food cooked on uncoated Cu cookware, Cu rich food, and smoke from burning Cu sulfate (Amorós et al., 2019; Royer and Sharman, 2023). Cu exposure may increase the release of proinflammatory cytokines and damage the structure of brain mitochondria, resulting in neurotoxicity (Kitazawa et al., 2016; Borchard et al., 2018). A cross-sectional European study found increased Cu in blood samples, was associated with higher CBCL internalizing symptoms in 6- to-11-year-old children (Maitre et al., 2021). Another European study found prenatal blood Cu levels to be negatively associated with SDQ externalizing scores in children ages 3–7 years old, suggesting decreased risk (Jedynak et al., 2021). The latter study noted their results should be interpreted with caution because there aren’t enough studies on Cu exposure and externalizing problems in children.

#### 3.2.4 Mercury

Elemental, organic, and inorganic forms of mercury (Hg) are neurotoxic to children and have been linked to neurodevelopmental and neurocognitive disorders (reviewed in Al-Osman et al., 2019). Hg bioaccumulates in the food chain, especially seafood, which is a main source of exposure for children and pregnant people (Bose-O’Reilly et al., 2010). One study explored the effects of Hg on behavioral health and found an association between increased maternal blood Hg levels and higher CBCL internalizing and externalizing scores in Korean toddlers aged 1–2 years (Kim et al., 2018). While this suggests that Hg may be implicated in abnormal internalizing and externalizing behaviors, there is a paucity of data. Other studies are needed to ascertain reproducibility across cohorts.

### 3.3 Endocrine-disrupting chemicals

Endocrine-disrupting chemicals (EDCs) are xenobiotics that mimic or inhibit hormones, and can lead to abnormal hormone signaling in tissues (Shoaff et al., 2019). EDCs are widely used in a myriad of products including, but not limited to, plastic containers, food packaging, personal care products, medical supplies, and building materials.

#### 3.3.1 Phthalates

High molecular weight phthalates are primarily used as plasticizers to increase flexibility and durability of plastics, while low molecular weight phthalates are used in cosmetics and pharmaceuticals (Chen et al., 2019). Phthalates easily leach into the environment and routes of exposure to children include inhalation, ingestion, and dermal absorption (Braun et al., 2013). Phthalates can also cross the placenta, resulting in prenatal exposure (Qian et al., 2020). Possible neurotoxic mechanisms for phthalates include altering steroid hormone concentrations, lipid metabolism, and disrupting neurotransmitters involved in the release of dopamine (Huang et al., 2019). In Canada, England-Mason et al. (2020) used MRI to examine brain white matter in pre-school children, and also conducted behavioral assessments. The authors found by neuroimaging and statistical modeling that increased prenatal exposure to high molecular weight phthalates was associated with changes in white matter microstructure in children (England-Mason et al., 2020). Specifically, increased mean diffusivity in brain regions that control affective function (i.e., mood) and perceptual processing. Further, the authors found indirect associations with increasing phthalate exposure during pregnancy and increased CBCL internalizing and externalizing scores. Together, these data suggest that the behavioral observations could be mediated by pthalate-induced structural changes in brain white matter (England-Mason et al., 2020).

In addition to the aforementioned study, several observational studies have also identified that increased phthalate exposure is correlated to behavioral outcomes. A U.S. birth cohort study found borderline statistical significance between increased prenatal exposure to multiple phthalates and temperament at 12- and 24-months (Singer et al., 2017). A Korean birth cohort study found a positive association between prenatal monoethyl phthalate (MEP) and CBCL internalizing scores in toddlers (Kim et al., 2018). In Taiwan, a birth cohort study assessed prenatal exposure to di (2-ethylhexyl) phthalate (DEHP) in 8-, 10-, and 14-year-old children (Chen et al., 2019). The authors found significant positive associations between prenatal urinary DEHP concentrations and increased CBCL scores in all of the test’s categories, except for somatic complaints. This indicates that children with higher phthalate exposure had behavioral problems in the following categories: withdrawn, anxious/depressed, social problems, thought problems, attention problems, delinquent behavior, aggressive behavior, internalizing problems, and externalizing problems. Children with higher DEHP exposure also had consistently higher overall CBCL scores (Chen et al., 2019). The authors noted that the median exposure quantified in their study was 4.54 μg/kg bw/day. This, in addition to previous epidemiological studies where DEHP exposure was associated with worsening behavioral symptoms, is well below the reference levels currently recommended by the EU and U.S., which is 50 and 20 μg/kg bw/day, respectively (Chen et al., 2019).

Another Taiwanese birth cohort study found associations between increasing maternal urinary DEHP metabolites and higher CBCL delinquent behavior and externalizing scores and increasing maternal urinary mono-2-ethylhexyl phthalate (MEHP) and higher CBCL internalizing and externalizing scores in children aged 8–14 years (Huang et al., 2019). The researchers also found a positive association between urinary monobenzyl phthalate (MBzP) at ages 2–8 years and CBCL scores for social problems at the age of 8–14 years (Huang et al., 2019). Colicino et al. (2021), studied di-2-ethylhexyl terephthalate (DEHT), a replacement for DEHP marketed as a less toxic alternative. In this Mexican birth cohort study, the authors linked higher maternal urinary DEHT levels with increased overall BASC-2 behavioral and depression scores in 4- to 6-year-old boys.

A French birth cohort study also reported that increased maternal urinary mono-n-butyl phthalate (MnBP) and MBzP during pregnancy were associated with increased SDQ internalizing behaviors in 3-year-old boys, however the incidence rate ratios were close to the null (Philippat et al., 2017). Jedynak et al. (2021) found prenatal urinary MnBP was associated with increased SDQ externalizing problems in 3-to7-year-old European children. In a cross-sectional design, Shoaff et al. (2019) collected spot urine from 15-year-old adolescents living near a Superfund site in the United States and assessed behavior using the BASC-2. The researchers observed a positive association between a sum of 11 antiandrogenic phthalate metabolites and increased maladaptive behaviors, such as externalizing behavior and developmental social disorders (Shoaff et al., 2019). Together, these data suggest that pthalate exposure during pregnancy is associated with a wide range of increased behavior problems in children, with ages ranging from infancy to adolescence.

In all, multiple studies conducted worldwide suggest that developmental phthalate exposure is correlated to behavioral deficits in children. Systematic reviews also suggest that increased developmental exposure to some phthalates is correlated with cognitive and psychomotor impairments, in addition to the behavioral issues reviewed here (Ejaredar et al., 2015; Zhang et al., 2019). While the etiology of these observations is unknown, phthalates are a diverse family of compounds with many metabolites. Additional research should be conducted to test the reproducibility of the epidemiological evidence cited herein.

#### 3.3.2 Polybrominated diphenyl ethers

Polybrominated diphenyl ethers (PBDEs) are flame retardants present in a variety consumer goods and materials, such as furniture, electronics, and car seats (Costa and Giordano, 2007). Children can inhale or ingest PBDEs from contaminated dust, as well as ingest these compounds from food (i.e., fatty fish) and breastmilk (Domingo, 2012; Malliari and Kalantzi, 2017). Data suggests that PBDE’s could negatively impact neurobehavior via several mechanisms including disrupting thyroid hormone action, inducing oxidative stress, altering brain protein expression, increasing neuronal apoptosis, altering cholinergic system responses, and disturbing neurotransmitter function (Costa and Giordano, 2007; Vuong et al., 2018; Strawn et al., 2022). Early life exposure to PBDEs, which can accumulate in tissues and cross the placenta, has been shown to negatively impact cognition and behavior in children (Costa and Giordano, 2007; Lam et al., 2017; Gibson et al., 2018; Strawn et al., 2022).

Three papers utilized data obtained from the Health Outcomes and Measures of the Environment (HOME) study, an ongoing U.S.-based birth cohort that assesses the health impact of early childhood exposures to environmental toxicants. Strawn et al. (2022) examined prenatal serum PBDE concentrations and found significant associations with increased anxiety symptoms in 12-year-olds, measured by the Screen for Child Anxiety Related Emotional Disorders. The strongest effects were observed for panic and separation anxiety (Strawn et al., 2022). Braun et al. (2017) found that higher prenatal serum BDE-47 exposure was associated with persistent increases in externalizing behaviors from ages 2 to 8, as quantified by BASC-2 scores. Vuong et al. (2017) assessed serum PBDE concentrations in 8-year-old children and saw a positive association between multiple PBDE congeners (BDE-28, BDE-47, BDE-153, ∑PBDEs) and externalizing symptoms. While these three papers provide great preliminary data, more research from different cohorts can help elucidate the relationship between BPDEs and adverse mental health symptoms.

#### 3.3.3 Bisphenols

A few studies investigated bisphenols, such as bisphenol A (BPA), an estrogenic polymer that is used to produce plastics (Ben-Jonathan and Steinmetz, 1998). In addition to prenatal exposure, children can be exposed to bisphenols from canned and packaged food, plastic baby and beverage bottles, and breast milk (Lakind and Naiman, 2011). Research suggests that BPA may disrupt the brain’s stress system (hypothalamic pituitary adrenal axis) and thus can increase symptoms of stress-related outcomes, such as anxiety and depression (Wiersielis et al., 2020). Several studies from years earlier than 2017 have linked prenatal and childhood BPA exposure to adverse behavior, anxiety, and depression (reviewed in Wiersielis et al., 2020).

In a China-based case-control study, researchers found that serum bisphenol AF exposure, a common BPA replacement, was associated with increased depressive symptoms in 7th grade students, with males being significantly more vulnerable than females (Zhang et al., 2022). A U.S. birth cohort observed a positive association between increased prenatal urinary BPA concentrations and greater BASC-2 externalizing scores in girls ages 2 through 8 years, but not boys (Braun et al., 2017). Using the HOME study cohort, Stacy et al. (2017) collected urinary BPA from pregnant people and children from the 2nd trimester through 8 years of age. The authors found that higher prenatal BPA concentrations were correlated with increased BASC-2 externalizing behavior in girls at 8 years of age. This same effect was not observed in boys. In contrast, higher BPA urinary concentrations was significantly associated with increasing externalizing behavior in boys, when both BPA and behavior were measured at 8-years of age. These studies suggest that BPA may elicit both temporal and sex-specific effects, similar to previous reports (Braun et al., 2009; Perera et al., 2016).

In the U.S., Shoaff et al. (2019) explored the cross-sectional relationship between 7 phenols (including bisphenol A, F, and S) in spot urine samples and adverse behavior in 15-year-old adolescents but produced null results. Another U.S. study observed an inverse association, indicating improved scores, between newborn BPA concentrations measured from dried blood spots and difficulties in SDQ prosocial behavior at 7 years old; however, this association was not significant when BPA concentrations were categorized in quartiles, making interpretation of these data difficult (Ghassabian et al., 2018). Jedynak et al. (2021) determined that increasing prenatal urinary BPA levels were associated with increased SDQ externalizing behaviors in European children ages 3–7 years. Assessing behavior in males with the SDQ in France, Philippat et al. (2017) found prenatal urinary BPA exposure was associated with increased internalizing behavior at 3 years old and increased externalizing behavior at 5 years old, as well as a positive association between triclosan and externalizing behavior at 3 years old.

Collectively, the bisphenol literature reviewed shows that abnormalities in externalizing and internalizing behaviors in children can be correlated to increased bisphenol exposure during pregnancy or childhood. However, the results of epidemiological studies correlate to factors such as the sex of the child and when bisphenols were measured. Additional studies are needed to confirm these observations, ideally with cohorts large enough in scale to permit stratification by sex and with bisphenol exposure measured sequentially at multiple times during development.

#### 3.3.4 Per- and polyfluoroalkyl substances

Per- and polyfluoroalkyl substances (PFAS) are a chemical class with several thousand different congeners, which are widely used in various consumer products from cookware to clothing (Glüge et al., 2020). Many PFAS are persistent in the environment and in humans and have been categorized as persistent organic pollutants (Baker and Knappe, 2022; Starnes et al., 2022). PFAS can be detected in the placenta, amniotic fluid, maternal and neonatal blood, and breastmilk, demonstrating that pregnant people and children have some body burden of PFAS (Vuong et al., 2021). These organohalogens can elicit endocrine disruption in animal models and may be neurotoxic via disruption of the thyroid system (Mariussen, 2012). PFAS may also induce oxidative stress in the brain, and alter dopaminergic signaling pathways (Mariussen, 2012; Salgado et al., 2016).

A U.S. prospective birth cohort study found that prenatal serum perfluorooctane sulfonic acid (PFOS), perfluorohexane sulfonic acid (PFHxS), and perfluorononanoic acid (PFNA) were associated with increased odds of externalizing behaviors, and PFHxS was associated with increased internalizing problems at ages 5 and 7 years (Vuong et al., 2021). Another U.S. prospective birth cohort study found that higher PFOS concentrations in dried blood spot from newborns was associated with increased odds of conduct and emotional problems at 7 years of age (Ghassabian et al., 2018). In a Danish prospective birth cohort, authors observed a positive relationship between prenatal plasma perfluorononanoic acid (PFNA) concentrations and externalizing behaviors at ages 7 and 11 years, as detected by the SDQ (Luo et al., 2020). Overall, the epidemiological studies reviewed here suggests that a developmental PFAS exposure may correlate to later abnormalities in both internalizing and externalizing behaviors. However, there are relatively few studies that examine the mental health consequences of PFAS exposure in children and adolescents. Given that ubiquity of PFAS exposure and their biological persistence, this relationship should be explored in future studies.

#### 3.3.5 Pesticides

Pesticides prevent, repel, and kill pests, and are integral to agriculture. Sources of pesticide exposure to children and pregnant people include contaminated food, water, indoor/outdoor air, household dust, and treated lawns (Roberts et al., 2012). Children who live in households close to agricultural sites, have household members working in agriculture, or who work in agriculture themselves, likely have higher exposure to various pesticides compared to the general population (Fenske et al., 2000; Lu et al., 2000).

Organochlorine pesticides, such as dichlorodiphenyltrichloroethane (DDT), were used heavily worldwide in the 1940s through 1960s. While they have been phased out of use in the U.S., organochlorines are still utilized in South America, Africa, and Asia for vector (i.e., mosquito) control. Nonetheless, these chemicals are persistent in the environment, and bioaccumulate in human tissue due to their lipophilic properties. They also likely cross the placenta, suggesting fetal exposure (Rosenquist et al., 2017). It is hypothesized that organochlorines dysregulate dopamine-mediated functions in animals, which is crucial to the limbic system, or the part of the brain that controls behavior and emotion (Rokoff et al., 2022). In Greenland and the Ukraine, Rosenquist et al. (2017) saw a positive association between a doubling of pre- and postnatal serum exposure to 1,1-dichloro-2,2-bis(*p*-chlorophenyl)-ethylene (*p,p*′-DDE), a metabolite of DDT, and odds of conduct problems in 5- and 7-year-olds. Rokoff et al. (2022) also looked at pre- and post-natal *p,p*′-DDE exposure and the effect on anxiety in 8- and 15-year-olds and found no association.

Organophosphate pesticides are applied widely in agriculture and work by inhibiting acetylcholinesterase (AChE) activity in insects. However, organophosphates also inhibit AChE activity in other species, and the cholinergic system plays a key role in mood regulation (Suarez-Lopez et al., 2021). A prospective cohort study in an Ecuadorian agricultural community saw an increase in adolescent (11–17 years) depression symptoms as AChE activity decreased, a relationship that was strongest at older ages and among female participants (Suarez-Lopez et al., 2021). The researchers did not detect any associations between AChE inhibition and anxiety symptoms (Suarez-Lopez et al., 2021).

Pyrethroid pesticides are widely utilized in homes, gardens, and agriculture; they are also used on pets and clothing to prevent ticks, and as vector control (Richardson et al., 2019). Pyrethroids can cross the blood-brain barrier and may alter various neurotransmitter systems and cause oxidative stress (Nasuti et al., 2007; Richardson et al., 2019). A longitudinal birth cohort study in the United States assessed prenatal exposure to pyrethroids and child behavior at ages at 4, 6, and 7–9 years (Furlong et al., 2017). Even with low detection frequencies, researchers found a positive association between detectable urinary levels of 3-phenoyxbenzoic acid (3-PBA) and cis-(2,2-dichlorovinyl)-2,2-dimethylcyclopropane-1-carboxylic acid (cis-DCCA) with increased BASC-2 internalizing and externalizing behaviors respectively. However, researchers advised interpreting the results with caution because exposure was categorized only as “detect” or “non-detect,” and not quantified. In all, the potential neurotoxicity of pesticides in humans is a known concern. However, there are few studies that specifically investigate if pesticide exposure is related to mental health concerns in children.

## 4 Discussion

The purpose of this review was to identify and summarize recent literature on environmental xenobiotic exposures and child mental health outcomes, specifically symptoms related to mood, anxiety, and behavioral disorders. The 29 studies included reveal that there is a growing body of literature demonstrating a potential relationship between increased exposure to pollutants like heavy metals, endocrine disrupting chemicals, and pesticides, with increased adverse mental health outcomes in children.

None of the studies in this review assessed the potential impact of environmental injustice. Low income and/or children of color living in communities disproportionately exposed to environmental pollution, often referred as “environmental justice,” or “overburdened” communities, may have increased vulnerability to the impact of chemical exposures on mental health outcomes. This may be due to increased chemical exposure(s), and/or an increased susceptibility to such environmental exposures due to additional psychological and physical stressors. For example, children in environmental justice communities may be exposed to higher levels of traumatic experiences, such as racial discrimination and environmental disasters (Jones Harden and Slopen, 2022). Furthermore, these communities may have less access to protective factors, such as greenspaces, healthy foods, quality healthcare and other neighborhood amenities (Reuben et al., 2022). It’s essential to understand the degree to which children in environmental justice communities are at increased risk and to what extent chemical exposures contribute to adverse physical health and mental health outcomes.

Potential opportunities to fill data gaps include conducting community based participatory research (CBPR) studies in communities where children and pregnant people have disproportionate exposure to chemically polluted sites (i.e., waste and wastewater facilities, industrial farming, metal working facilities, manufacturers, and oil and gas refineries), and/or xenobiotic exposure from sources like contaminated seafood, lead paint, unsafe water, and certain personal care products.

Along these lines, more research is needed to understand the cumulative impact of prenatal and childhood exposure to chemical and non-chemical stressors (i.e., psychosocial stress) on mental health outcomes, as well as the potential impact of protective factors, For example, Maitre et al. (2021) used an exposome approach to assess chemical and non-chemical environmental stressors and found longer sleep duration, higher family social capital, and a healthy diet during childhood to be protective against behavioral symptoms. Mental health outcomes could also be included in policy and regulatory cumulative impact/risk frameworks, especially given their potential economic burden (Torio et al., 2015; Trautmann et al., 2016; Tkacz and Brady, 2021).

Furthermore, a chemical exposure rarely occurs in isolation, and instead, complex mixtures more accurately represent a human exposure scenario. Two studies included in this review explored chemical mixtures. Horton et al. (2018) found a metal mixture of manganese, zinc and lead was associated with increased anxiety symptoms, with the mixture driven by Mn at 0–8 months and Pb at 8–12 months. When looking at prenatal exposure to a mixture of organochlorines and metals in 8- and 15-year-olds, Rokoff et al. (2022) saw null results. In addition, there have been other recent studies exploring the relationship between chemical mixtures and mental health related outcomes in children (Cowell et al., 2021; de Water et al., 2022). More research to understand chemical interactions in humans is critical, especially during pregnancy and development (Kim et al., 2018).

Hypothesized neurotoxic mechanisms that can translate to adverse mental health outcomes are discussed for each chemical throughout this review. However, only a few studies explored potential mechanisms. There is a need for mechanistic studies to confirm these hypotheses and preliminary results, including additional fMRI and MRI studies showing which regions of the brain may be affected by environmental exposures, as well as hypothesis-driven animal studies. It is difficult for prospective cohort studies to show causation between a chemical exposure and a health effect. As such, if the observations identified in these human studies could be repeated and further explored in an animal model, it would add further weight of evidence linking an exposure to a mental health outcome. In addition, 8 studies in this review saw statistically significant effects, but only in one sex. Future work is also needed to understand sex differences in neurological development, to help explain sex-specific outcomes.

Most studies included in this review are cohorts comprised of mother-child pairs; this is crucial, as it is well established that fetal and early postnatal development is a period of vulnerability to environmental exposures (Bellinger, 2013). However, some of the data reviewed suggest that chemical exposures during later childhood is also linked to mental health outcomes. Therefore, additional longitudinal birth cohort studies would allow for multiple exposure assessments during pregnancy, infancy, and childhood, providing a unique opportunity to explore windows of vulnerability to chemical exposures. Many mental health disorders are not diagnosed until adolescence (Solmi et al., 2022), thus evaluating children in epidemiological studies during this developmental period, instead of infancy and early childhood, may reveal stronger associations between a chemical exposure and neurological health.

Many of the papers in this review controlled for population variables like sociodemographic factors, birth conditions, and family history of mental illness. Future areas of study could explore potential interactions between these risk factors and chemical exposures of interest. And finally, existing national surveys could be expanded to help fill data gaps. For example, the National Survey of Children’s Health currently includes a mental health questionnaire but lacks any environmental exposure data. While an exposure would not be quantified due to the lack of biological sampling, researchers could infer exposure risk based on a participant’s geographical location if this information was made available. In all, recruiting large cohorts of mother-child dyads for extended observational studies, and leveraging existing tools for environmental health research, could further this important area of study.

## 5 Conclusion

Understanding the impact of environmental chemicals on children’s mental health is critical to promotion of health, wellbeing, and the prevention of disease (Thoma et al., 2021; Reuben et al., 2022). Due to the complex nature of both environmental xenobiotic exposures and mental health outcomes, it is difficult to establish causality with epidemiology studies alone. Unfortunately, the existing data are too limited to formally determine how individual chemicals may influence the development of specific mental health outcomes, though research in this area are steadily growing. With increased studies, this could permit data synthesis through tools like systematic review and metanalysis, and lead to sound scientific conclusions. Not only could this better inform policy and regulations to minimize any potential adverse effects of environmental chemicals, but it could also lead to informed risk management decisions for individuals, communities, and medical professionals. Given the burden of mental health disorders on children’s health, wellbeing, and overall life trajectory, it is essential to identify and take action to address the environmental risks that may increase the development of these disorders.

.
